# The Potential Role of *As-sumo-1* in the Embryonic Diapause Process and Early Embryo Development of *Artemia sinica*

**DOI:** 10.1371/journal.pone.0085343

**Published:** 2014-01-03

**Authors:** Bing Chu, Feng Yao, Cheng Cheng, Yang Wu, Yanli Mei, Xuejie Li, Yan Liu, Peisheng Wang, Lin Hou, Xiangyang Zou

**Affiliations:** 1 College of Life Sciences, Liaoning Normal University, Dalian, PR China; 2 Department of Biology, Dalian Medical University, Dalian, PR China; State Key Laboratory of Reproductive Biology, Institute of Zoology, Chinese Academy of Sciences, China

## Abstract

During embryonic development of *Artemia sinica*, environmental stresses induce the embryo diapause phenomenon, required to resist apoptosis and regulate cell cycle activity. The small ubiquitin-related modifier-1 (SUMO), a reversible post-translational protein modifier, plays an important role in embryo development. SUMO regulates multiple cellular processes, including development and other biological processes. The molecular mechanism of diapause, diapause termination and the role of *As-sumo-1* in this processes and in early embryo development of *Artemia sinica* still remains unknown. In this study, the complete cDNA sequences of the *sumo-1* homolog, *sumo ligase* homolog, *caspase-1* homolog and *cyclin B* homolog from *Artemia sinica* were cloned. The mRNA expression patterns of *As-sumo-1*, *sumo ligase*, *caspase-1*, *cyclin B* and the location of *As-sumo-1* were investigated. SUMO-1, p53, Mdm2, Caspase-1, Cyclin B and Cyclin E proteins were analyzed during different developmental stages of the embryo of *A. sinica*. Small interfering RNA (siRNA) was used to verify the function of *sumo-1* in *A. sinica.* The full-length cDNA of *As-sumo-1* was 476 bp, encoding a 92 amino acid protein. The *As-caspases-1* cDNA was 966 bp, encoding a 245 amino-acid protein. The *As-sumo ligase* cDNA was 1556 bp encoding, a 343 amino acid protein, and the *cyclin B* cDNA was 739 bp, encoding a 133 amino acid protein. The expressions of *As-sumo-1*, *As-caspase-1* and *As-cyclin B* were highest at the 10 h stage of embryonic development, and *As-sumo ligase* showed its highest expression at 0 h. The expression of *As*-SUMO-1 showed no tissue or organ specificity. Western blotting showed high expression of *As*-SUMO-1, p53, Mdm2, Caspase-1, Cyclin B and Cyclin E at the 10 h stage. The siRNA caused abnormal development of the embryo, with increased malformation and mortality. *As*-SUMO-1 is a crucial regulation and modification protein resumption of embryonic diapause and early embryo development of *A. sinica*.

## Introduction

*Artemia sinica*, a small aquatic crustacean, lives in the hyperosmotic environment of salt pools and salt lakes in China [1]. *A. sinic*a, as an experimental material, is widely used in many fields including physiology, developmental biology, evolution and ecology [2]. *Artemia* have a peculiar diapause process during embryo development, which has received increasing attention from scientists studying the causes and molecular mechanisms of diapause termination of embryo development, especially the molecular mechanism of resistance to apoptosis and regulation of cell cycle activity in *Artemia* embryos.

Post-translational modifications are involved many cellular processes, such as signal transduction, protein localization and the cell cycle [3]. Phosphorylation, methylation and other modifications by small molecules act as post-translational modifiers. One of the best known modifiers is ubiquitin, which mediates degradation of target proteins by the 26S proteasome [4]. A number of small proteins, classified as ubiquitin-like modifiers (Ubls), have been identified to be covalently attached to target proteins in a similar manner to ubiquitylation. The small ubiquitin-related modifier (SUMO) was defined as a post-translational modifier following the identification of the first SUMO gene (SMT3) and the first substrate (RanGAP1, Ran GTPase-activating protein 1) [5], [6].

Invertebrates only have a single SUMO gene, while plants and vertebrates have several [7]. The sumoylation pathway resembles ubiquitin conjugation, but the enzymes catalyzing the two processes are distinct, although they share similarities [8]–[10]. ATP activates SUMO-1, as in the process of ubiquitylation. SUMO conjugation is initiated via a cascade of enzymatic reactions consisting of E1, E2 and E3 enzymes. The SUMO-activating enzyme (E1: a heterodimer between Aos1 and Uba2) initiates the process by first catalyzing adenylation of the SUMO C-terminus. SUMO is subsequently transferred to the active site cysteine of the SUMO E2 conjugating enzyme, Ubc9. Ultimately, the modifier is ligated to the ε-amino group of a lysine on the substrate, with or without the help of the Sumo-pathway-specific E3 protein [11], [12]. SUMO conjugation frequently occurs at a consensus sequence that is present on most, but not all targets, designated ψKxD/E [13]. SUMO modification is a dynamic, reversible process, and removal of SUMO is carried out by SUMO-specific proteases that specifically cleave at the C-terminus of SUMO [14]–[16].

Many studies of *sumo-1* have focused on human or model animals; however, the expression pattern, distribution and the role of *sumo-1* in post-diapause and early embryo development of *A. sinica* remain unknown. In the present study, cDNAs representing the *As-sumo-1*, *As-sumo ligase*, *As-caspase-1* and *As-cyclin B* genes were cloned by rapid amplification of cDNA ends (RACE). The expression patterns and expression location of *As-sumo-1* during development of *A. sinica* was investigated by real-time PCR and immunochemistry. The expression level of SUMO-1, p53, Mdm2, Caspase-1, Cyclin B and Cyclin E proteins during different developmental stages were analyzed by western blotting. siRNA depletion of *As-sumo-1* was carried out to further investigate the functions of *As-sumo-1* in postdiapause and early embryo development of *A. sinica*. Our aim was to further our understanding of the function of *As-sumo-1* and the other proteins in regulation and modification of the cell cycle and apoptosis during postdiapause and in early embryo developmental stages of *A. sinica*.

## Materials and Methods

### Animal and Sampling

No specific permits were required for our samples of *Artemia* cysts collected and field studies. The location was not privately-owned or protected in any way, and the field studies also did not involve endangered or protected species. We confirm that the salt lake and land we conducted our study on was not privately owned or government protected.

*A. sinica* cysts were collected from the Salt Lake of Yuncheng in Shanxi Province (China). The cysts (about 50 mg) were maintained in filtered seawater and hatched at 28°C, at a salinity level of 28 practical salinity units (PSU), under a light intensity of 1,000 lx. The samples were collected at different time points (0 h, 5 h, 10 h, 15 h, 20 h, 40 h, 3 d, 5 d) for subsequent experiments. 0 h represents the gastrula stage of *A.* cysts, and this stage belong to post diapause stage; 5 h, 10 h, 15 h, embryonic stage; 20 h and 40 h, nauplius stage; 3 d, metanauplius larva stage; and 5 d, 7 d, 10 d, pseudoadult stage.

### Full Length cDNA Cloning of *As-sumo-1, As-sumo Ligase, As-caspase-1, As-cyclin B*

To prepare cDNA templates for PCR amplifications, total RNA was extracted using Trizol A+(Tiangen, Bejing, China) according to the manufacturer’s instructions, followed by reverse transcription using an oligo (dT) primer and MLV reverse transcriptase (Takara, Dalian, China), also following the manufacturer’s protocol. Expressed sequence tags (ESTs) of *sumo-1*, *sumo ligase*, *caspase-1* and *cyclin B* of *A. franciscana* were obtained from GenBank and used to design primers using Primer 5.0. All genes-specific primers used for cloning *sumo-1*, *sumo ligase*, *caspase-1*, *cyclin B* were synthesized by TaKaRa and are shown in Table 1.

**Table 1:** Oligonucleotide primers used in the study.

Primer	Sequence (5′–3′)	Direction	Program
*sumo-1*-F	GCTAATAGTAGTTGGACA	Forward	EST
*sumo*-*1*-R	CAAGCGTAAGATACAGGA	Reverse	EST
*sumo ligase*-F	TGCGATTTGCTTTGCTTGA	Forward	EST
*sumo ligase*-R	TCCATAGGTCTTCTTCCTTTCTT	Reverse	EST
*caspase*-*1*-F	CCCGAAATACTCTGGTCT	Forward	EST
*caspase*-*1*-R	GATTCATAGTCAAAGGCAAC	Reverse	EST
*cyclin B*–F	TACAGCAGCGGGAACAGT	Forward	EST
*cyclin B*–R	CTTCAATCAGCCAATCTA	Reverse	EST
3′-*sumo-1*	GAGGAGACGCCCAAACAACT	Forward	RACE
5′-*sumo-1*	CGCCCACTCGTTCACTGTATGACTTT	Reverse	RACE
3′-*sumo ligase* GSP1	CCCAAAAGACCCTCACTACC	Forward	RACE
3′-*sumo ligase* GSP2	GCCTGATTCTAATGCTTTCG	Forward	RACE
5′-*sumo ligase* GSP1	TATCAACTGGTAGTGAGGGTCT	Reverse	RACE
5′-*sumo ligase* GSP2	CATTTTGGGACTCTACCAGGCT	Reverse	RACE
3′- *caspase*-*1* GSP1	ACTCGGGTTCGTTCTCCTAC	Forward	RACE
3′- *caspase*-*1* GSP2	TGGTTTGTTCAAGCCCTGTG	Forward	RACE
5′- *caspase*-*1* GSP1	GTTTCTGTATTGCCACGGAGGGT	Reverse	RACE
5′- *caspase*-*1* GSP2	TGCCACGGAGGGTAAGAAGAGTT	Reverse	RACE
3′- *cyclin B* GSP1	ACAGCAGCGGGAACAGTGAG	Forward	RACE
3′- *cyclin B* GSP2	AAAGTCGGAACAACTCAACG	Forward	RACE
5′- *cyclin B*	TGTTCCGACTTTTTTGACATCCTTC	Reverse	RACE
*sumo*-*1* 5′ primer	GATGGTAGGAGAATCAATG	Reverse	Real time
*sumo*-*1* 3′ primer	CAAGCGTAAGATACAGGAG	Forward	Real time
*sumo ligase* 5′ primer	GATGTGTCCAGTGTGTGA	Reverse	Real time
*sumo ligase* 3′ primer	ATTTAGGTTCCCAAGAGC	Forward	Real time
*caspase-1* 5′ primer	TTCTTACCCTCCGTGGCA	Reverse	Real time
*caspase-1* 3′ primer	TCCGTTGGTTGTGTTTCG	Forward	Real time
*cyclin B* 5′ primer	AGCAGCGGGAACAGTGAG	Reverse	Real time
*cyclin B* 3′ primer	GAGGGGGGCTTTGGAATA	Forward	Real time
*β-actin* 3′primer	AGCGGTTGCCATTTATTGTT	Forward	Real time
*β-actin* 5′primer	GGTCGTGACTTGACGGACTATAT	Reverse	Real time
*sumo-1*-Pro-F	CGGAATTC ATGTCTGATGAAAACAAAGATGCTG	Forward	*As-sumo-1* ORF
*sumo*-*1*-Pro–R	CCGCTCGAG TTAAAAACCTCCCGTTTGTTCCT	Reverse	*As-sumo-1* ORF
Sense siRNA	AAGTCATACAGTGAACGAGTGUU	Forward	siRNA
Anti siRNA	CACTCGTTCACTGTATGACUU	Reverse	siRNA

The PCR conditions were as follows: initial incubation at 94°C for 3 min; followed by 30 cycles of denaturation at 94°C for 30 s, annealing at 47.5°C for 30 s and elongation at 72°C for 1 min; with a final incubation at 72°C for 10 min. The PCR products were separated on 1.0% agarose/TAE gels and sequenced by Takara.

RACE was performed according to the Smart RACE cDNA Amplification Kit (ClonTech, Dalian, China) and 3′ Full RACE Amplification Kit (Takara). The RACE primers were synthesized by TaKaRa and are shown in Table 1. The target PCR products were cloned into vector pMD18-T (TaKaRa) and sequenced by TaKaRa. The RACE–PCR products were purified using gel electrophoreses and cloned into vector pMD-19T (Takara) for sequencing. The 3′- and 5′- terminal fragments were spliced together using DNAman (Lynnon Biosoft) to yield the full length cDNA.

### Real Time PCR and Bioinformatics Analysis

mRNA samples were extracted from *A. sinica* at different time points (0 h, 5 h, 10 h, 15 h, 20 h and 40 h; 3, 5 d) and as templates for cDNA synthesis using a Two-step Reverse Transcription Kit (Takara). TaKaRa synthesized the primers for real-time PCR (Table 1). Real-time PCR was performed in triplicate for each sample using SYBR® Premix Ex TaqTM (Takara) and the Takara TP800 Detection System. Each reaction (25 µl) was performed with 2 × SYBR® Premix Ex TaqTM and 10 µM of each primer in a 100 µl tube. The reaction conditions were as follows: initial denaturation step at 95°C for 30s, and 40 cycles of amplification (95°C for 5 s, 57°C for 30 s, 95°C for 15 s, 60°C for 30 s, 95°C for 15 s). The specific primers used for PCR of *As-sumo-1*, *sumo ligase*, *caspase-1*, *cyclin B* and *β-actin* are shown in Table 1. The *A. sinica β-actin* gene was used to normalize the starting quantity of each RNA sample. Data obtained from real-time quantitative PCR analysis were analyzed using the LSD t-test in SPSS 16.0 to determine differences in the mean values between treatments and control group; the significance threshold was *P<0.05*.

Bioinformatics analysis of *sumo-1*, *sumo ligase*, *caspase-1* and *cyclin B* was performed using software programs on the NCBI website (http://www.ncbi.nlm.nih.gov). The full length cDNAs were subjected to bioinformatics analysis using an ORF finder tool (http://www.ncbi.nlm.nih.gov/gorf/gorf.html). Sequence alignment and phylogenetic analysis was performed using ClustalX 2.0, MEGA4.0 and the NCBI online service (http://www.ncbi.nlm.nih.gov/). The molecular weight and pI were calculated using Compute pI/Mw (http://us.expasy.org/tools/pi_tool.html). Signal peptides were predicted using the SignalP 3.0 server (http://www.cbs.dtu.dk/services/SignalP/). The NetPhos 2.0 Server (http://www.cbs.dtu.dk/services/NetPhos/) was used to determine the phosphorylation sites. The TMpred server (http://www.ch.embnet.org/software/TMPREDform.html) was used to detect transmembrane regions. The phylogenetic tree was constructed by the Neighbor-joining (NJ) method using ClustalX 2.0 and MEGA 4.1 software. The statistical significance of groups within the phylogenetic tree was evaluated using the bootstrap method with 1,000 replications.

### Prokaryotic Expression of As-SUMO-1 Like Protein

#### Cloning the *As-sumo-1* ORF

The primers *sumo-1*-Pro-F and *sumo-1*-Pro-R, which included EcoRI and XhoI restriction endonuclease recognition sites (underlined) at their 5′ (Table 1), were designed with Primer Premier 5.0, based on the *As-sumo-1* full-length sequence (GenBank accession: JQ855852.1), and synthesized by TaKaRa (Dalian, China). The PCR reaction conditions were stated above.

#### Construction of the cloning and expression vectors

The obtained PCR products were cloned into the pMD19-T Simple Vector (TaKaRa); the recombinant plasmid was termed pMD19-T-SUMO-1. Both pMD19-T-SUMO-1 and the pET-30a expression vector were digested with EcoRI and XhoI, and ligated to form recombinant plasmid pET-30a-SUMO-1. TaKaRa (Dalian, China) sequenced the two recombinant plasmids. DNAstar analyzed the molecular mass and isoelectric point of the recombinant proteins.

#### Expression, soluble detection and purification of the *As*-SUMO-1 recombinant protein

This recombinant *As*-SUMO-1 protein was expressed in *E. coli* BL21 (DE3) using four induction conditions: 1 mM IPTG for 3 h at 37°C, 1 mM IPTG for 3 h at 30°C, 0.25 mM IPTG for 3 h at 37°C, and 0.25 mM IPTG for 3 h at 30°C. Cells were collected by centrifugation at 13,000 g for 5 min at 4°C. The supernatants were discarded, and the cell pellets were washed twice with phosphate buffered saline (PBS) before being resuspended in PBS. The samples were boiled for 8 min after adding one volume of 2× SDS–PAGE loading buffer. The best of the four induction conditions for large-scale purification by ultrasonication was basing on SDS–PAGE analysis of the supernatant and cell pellet.

The recombinant SUMO-1 protein was expressed in a 1 L culture of *E. coli* BL21 (DE3), induced with 0.25 mM IPTG at 30°C for 3 h. Cells were collected by centrifugation at 7,000 rpm for 10 min at 4°C and the sediment was resuspended in equilibration buffer containing 20 mM Tris–HCl (pH 8.5), 150 mM NaCl and 20 mM imidazole, before being lysed by ultrasonication. Purification of the *As*-SUMO-1 recombinant protein was accomplished with a HisTrapTM FF crude (GE Healthcare), following the supplier’s protocol. The only differences in the washing buffer, elution buffer, and equilibration buffer were the imidazole concentrations: 20 mM, 40 mM, 60 mM,80 mM,100 mM and 300 mM, respectively. The protein was dialyzed into 20 mM Tris-HCl and then freeze dried by lyophilizer (Japan).

#### Production of polyclonal antibodies

All studies involving rabbits were approved by Animal Care and Use Committee of Dalian Medical University, Dalian, Liaoning, China. Eight-week-old female New Zealand rabbits (Specific pathogen Free, SPF) were obtained from Experiment Animal Center of Dalian Medical University. All experimental procedures were conducted in conformity with institutional guidelines for the care and use of laboratory animals. Polyclonal antibodies directed against the *As*-SUMO-1 recombinant protein were prepared in a New Zealand Rabbit. Before the first injection, we extract 1 ml blood from the ear marginal vein of the rabbit as a negative control. Rabbits were immunized every ten days by back subcutaneous multipoint injection. The purified protein (600 µg/ml) was emulsified in an equal volume of Freund’s complete adjuvant for the first immunization. For the next three immunizations, the purified protein (300 µg/ml) was emulsified with an equal volume of Freund’s incomplete adjuvant. The antiserum was collected by centrifugation at 13,000 g for 5 min, and ELISA was used to check the protein concentration. Western blotting was used to determine the specificity of the antibody to the purified protein. Recombinant *As*-SUMO-1 protein were detected using specific His-tag antibodies by Western blot.

### Western Blotting

The samples were collected at different developmental stages (0, 5, 10, 15, 20, and 40 h, 3 d) and total proteins were extracted from each sample with RIPA Lysis Buffer and quantified using the Bradford method. Each protein sample (70 µg) was fractionated by SDS–PAGE and transferred to PVDF membranes. The membranes were blocked in PBST containing 5% skim milk for 1 h at room temperature. Rabbit anti-*As*-SUMO-1 polyclonal antibody and GAPDH antibody were diluted 1∶200 and 1∶1000, respectively, with PBST, and incubated with the membranes at 4°C overnight. The membranes were washed with PBST (3×10 min), and then incubated with HRP-conjugated goat anti-rabbit IgG antibody for 1 h at 37°C, followed by washing with PBST three times and PBS once. The membrane was incubated with ECL reagent (Transgen, Beijing, China) and exposed to an X-ray film in the darkroom. The films were photographed and then analyzed using Image J software (National Institutes of Health) using the image gray scale analysis method in the Image J documentation, which can compare the density (equivalent to the intensity) of the bands on the western blot. The data was used to build column charts. The expression intensities of *As*-SUMO-1-specific bands were normalized against the GAPDH-specific bands. Except *As*-SUMO-1 polyclonal antibody, the other antibodies were bought from Santa Cruz Biotechnology (Shanghai) Co., Ltd according to the homology of N terminal and C terminal among other proteins. The homology were all more than 60%. The western blot steps of other proteins were similar to *As-*SUMO-1.

### Immunohistochemistry of As-SUMO-1

The nauplii were collected at different developmental stages (0, 5, 10, 15, 20, and 40 h, 3 d and 5 d), rinsed with distilled water and fixed in 4% paraformaldehyde solution for 5 h at 4°C. Infiltration in dimethylbenzene (2×10 min) and absolute ethyl alcohol (2×5 min), was followed by 95%, 80%, 70% and 50% ethanol (5 min each). The sections were then washed once with running water for 10 min, and with PBS (3×3 min), placed at room temperature for 15 min after adding endogenous catalase blockers, washed with PBS (3×3 min), before being blocked with 0.5% BSA for 20 min. The rabbit anti-*As*-SUMO-1 polyclonal antibody (diluted 1∶100 with PBS) was added and the sections were incubated overnight at 4°C. The sections were then washed with PBS (3×3 min), incubated with HRP-conjugated goat anti-rabbit IgG antibody for 1 h and washed with PBS (3×3 min). The sections were incubated at room temperature for 15 min with streptavidin peroxidase. Samples were stained with diaminobenzidine after washing with PBS and counterstained with hematoxylin, infiltrated in 70% ethanol for 5 min, 95% ethanol and then absolute ethyl alcohol (2×5 min), and finally dimethylbenzene (2×10 min). Sections were immobilized with resinene and examined under the microscope.

### siRNA Assay

RNA oligonucleotides for *sumo-1* were designed by an on-line system from the full length cDNA of *As-sumo-1* and synthesized by Takara (Table 1). The 0 h samples were exuviated using 50% NaClO. For electroporation, samples were suspended in electroporation buffer containing the same amount of dsRNA as used for soaking incubation. The sample suspensions were electroporated at 400 V for 1s after adding 4 mM dsRNA using EC100. Seawater was then added and the embryos were incubated in a constant temperature incubator for up to 0 h, 5 h, 10 h, 15 h and 20 h. No oligonucleotide controls were processed identically. Total RNA was extracted from siRNA treated larvae of 0 h, 5 h, 10 h, 15 h, 20 h and controls using Trizol (Tiangen, China) and subjected to real time PCR as detailed above.

## Results

### Cloning and Bioinformatic Analysis of *As-sumo-1*, *As-caspase-1*, *As-sumo Ligase*, *As-Cyclin B*

**Figure 1:**
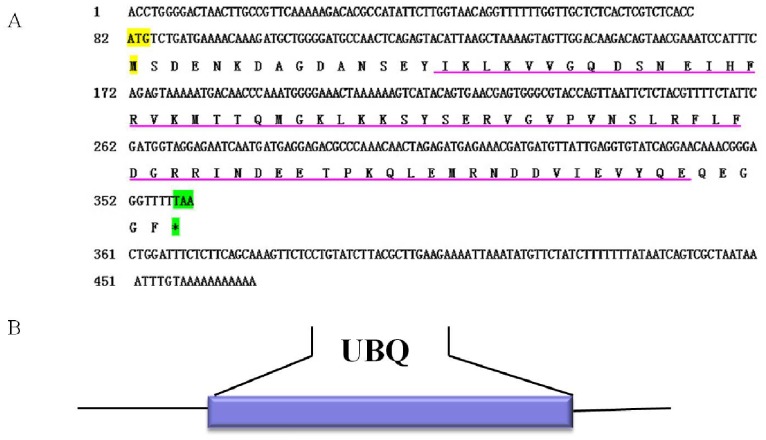
Nucleotide and deduced amino acid sequences of *As-sumo-1* and putative protein domain. (A) Sequence analysis of the cDNA and predicted peptide sequences of *As-sumo-1*. The start codon is indicated in yellow; the stop codon is indicated in green. The region defined by a straight purple line is the UBQ domain. (B) Result of domain analysis of putative As-SUMO-1 protein. The mature putative protein includes a UBQ domain.

**Figure 2:**
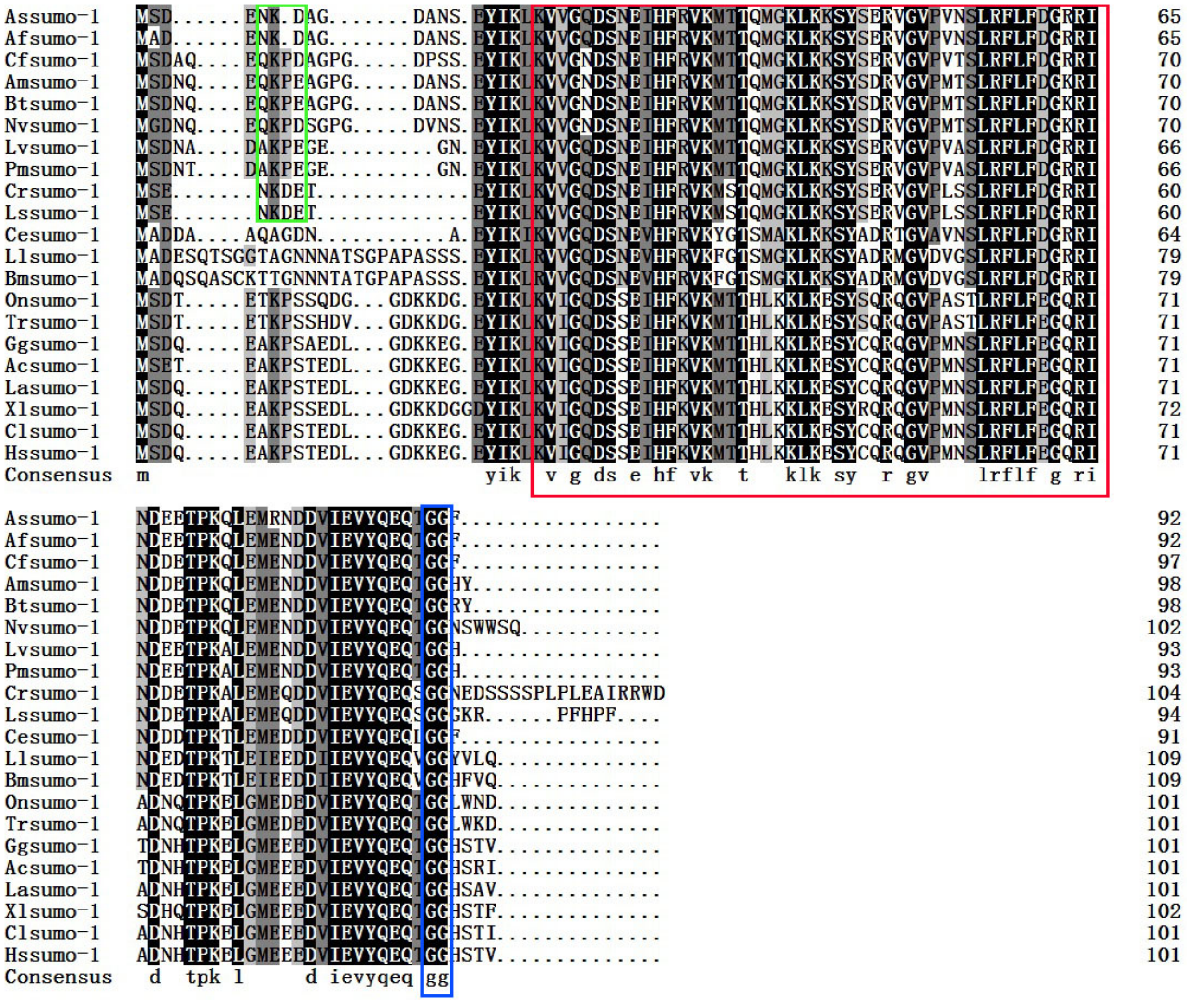
Multiple sequence alignment of the As-SUMO-1 protein. Sequence alignment of known *sumo-1* isoforms from 20 species. Black shading indicates identical amino-acid residues. Gray shading indicates less conserved residues. Pale gray shading indicates somewhat similar residues. The sequences and the accession numbers of *sumo-1* are as follows: Assumo-1, *Artemia sinica*, AFH36133.1; Afsumo-1, *Artemia franciscana*, ABQ41279.1; Cfsumo-1, *Coptotermes formosanus* AGM32544.1; Amsumo-1, *Apis mellifera* XP_392826.1; Btsumo-1, *Bombus terrestris* sumo-1, XP_003394722.1; Nvsumo-1, *Nasonia vitripennis* XP_001607301.1; Lvsumo-1, *Litopenaeus vannamei*, ACR56783.1; Pmsumo-1, *Penaeus monodon* sumo-1, ACD13593.1; Ccsumo-1, *Caligus clemensi*, ACO15528.1; Lssumo-1, *Lepeophtheirus salmonis*, ACO12186.1; Cesumo-1, Caenorhabditis elegans, NP_490842.1; Llsumo-1, *Loa loa*, XP_003137066.1; Bmsumo-1, *Brugia malayi*, XP_001900504.1; Onsumo-1, *Oreochromis niloticus*, XP_003446582.1; Trsumo-1, *Takifugu rubripes* sumo-1, XP_003966620.1; Ggsumo-1, *Gallus gallus*,NP_989466.1; Acsumo-1, *Anolis carolinensis* XP_003223593.1; Lasumo-1, *Loxodonta africana*, XP_003406178.1; Xlsumo-1, *Xenopus laevis*, NP_001083717.1; Clsumo-1, *Canis lupus familiaris*, NP_001239192.1; Hssumo-1, *Homo sapiens* sumo-1, NP_003343.1. The ubiquitin domain is boxed in red and the C-terminal double Gly-Gly residues for conjugation are boxed in blue. The sumoylation consensus ψ KXE/D motif is boxed in green. In ψ KXE/D,‘ ψ’ represents a hydrophobic amino acid residue and ‘ X ’ any residue.

**Figure 3:**
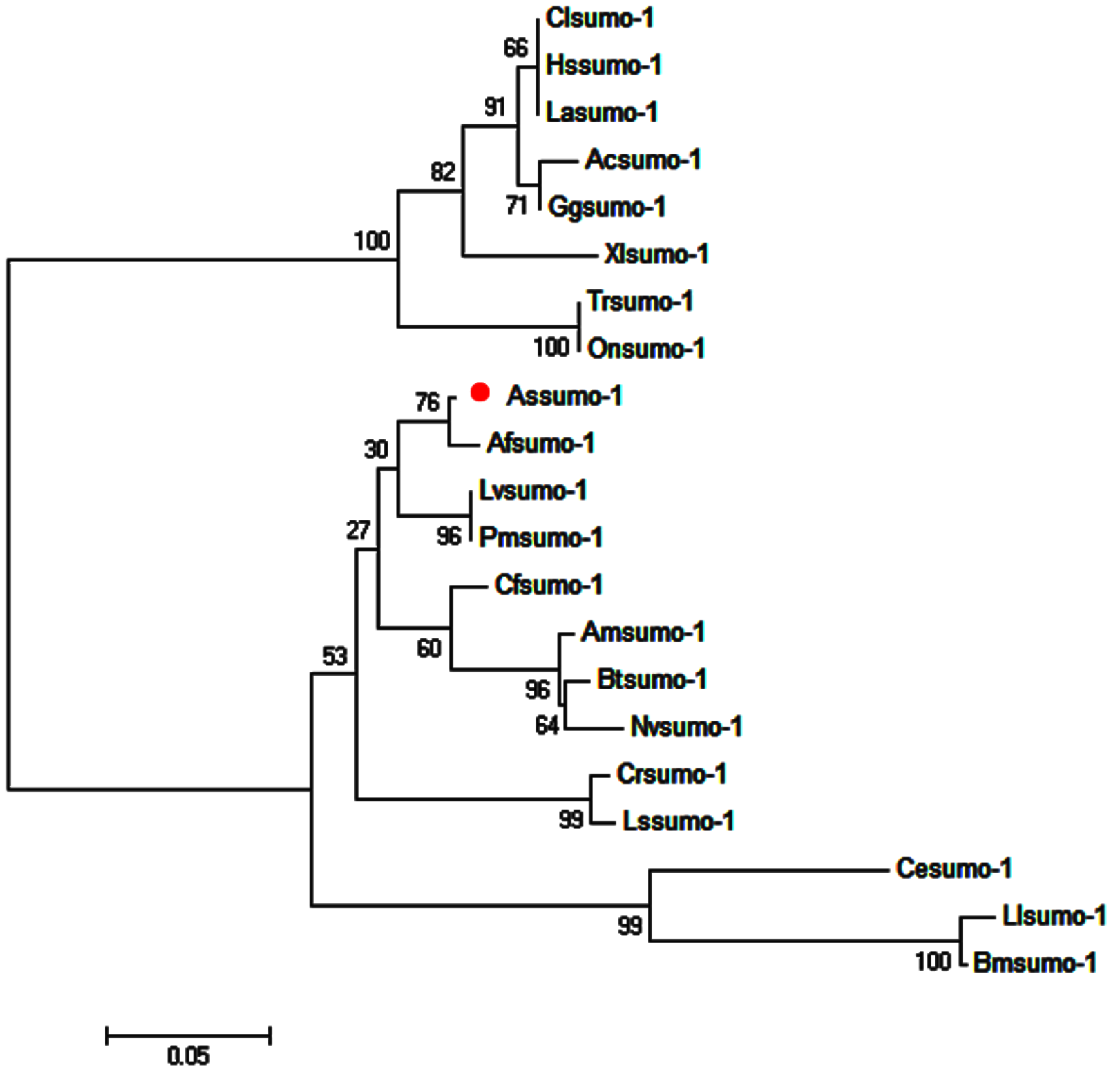
The neighbor-joining phylogenetic analysis of SUMO-1 protein. Phylogenetic tree of aligned amino-acid sequences of *sumo-1* from *Artemia sinica* and 20 other species. A neighbor-joining phylogenetic tree was constructed using *sumo-1* sequences from *A. sinica* and 20 other sequences from GenBank, using the sequence analysis tool MEGA 4.1. The sequences and their accession numbers are indicated in the legend of Fig. 2. A circle (•) indicates sumo-1 from *A. sinica*.

The full-length cDNA of *sumo-1* gene was in Fig. 1. SignalP3.0 analysis showed that *As-*SUMO-1 has no signal peptide. Secondary structure prediction demonstrated that the protein secondary structure comprised an Alpha helix (Hh; 7.61%), an Extended strand (Ee; 25%) and a Random coil (Cc; 67.39%). The protein has no transmembrane domain. Hydrophobicity analysis indicated that SUMO-1 is mostly hydrophilic. SUMO-1 was predicted to have three serine phosphorylation sites, a threonine phosphorylation site and a tyrosine phosphorylation site. Bioinformatic analysis suggested that SUMO-1 had the highest sequence homology (98% identity) with SUMO-1 from *Artemia franciscana* (Fig. 2). The SUMO-1 sequences of 21 species were selected to construct a phylogenetic tree (Bootstrapping = 1000). The phylogenetic tree (Fig. 3) showed three main clusters: vertebrates, arthropods and nematodes, with *As*-*sumo-*1 clustered within the arthropods. The relationships displayed in the phylogenetic tree corresponded with their taxonomic classification.

**Figure 4:**
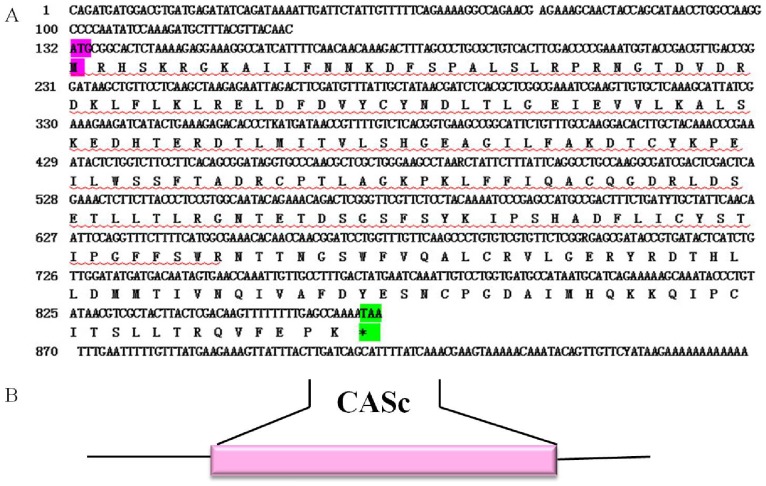
Nucleotide and deduced amino acid sequences of *As-caspase-1* and putative protein domain. A. Sequence analysis of the cDNA and predicted peptide sequences of *As-caspase-1*. The start codon is indicated in purple; the stop codon is indicated in green. The region defined by a wavy red line is the CASc domain. B. Result of domain analysis of putative As-Caspase-1 protein. The mature putative protein includes a CASc domain.

**Figure 5:**
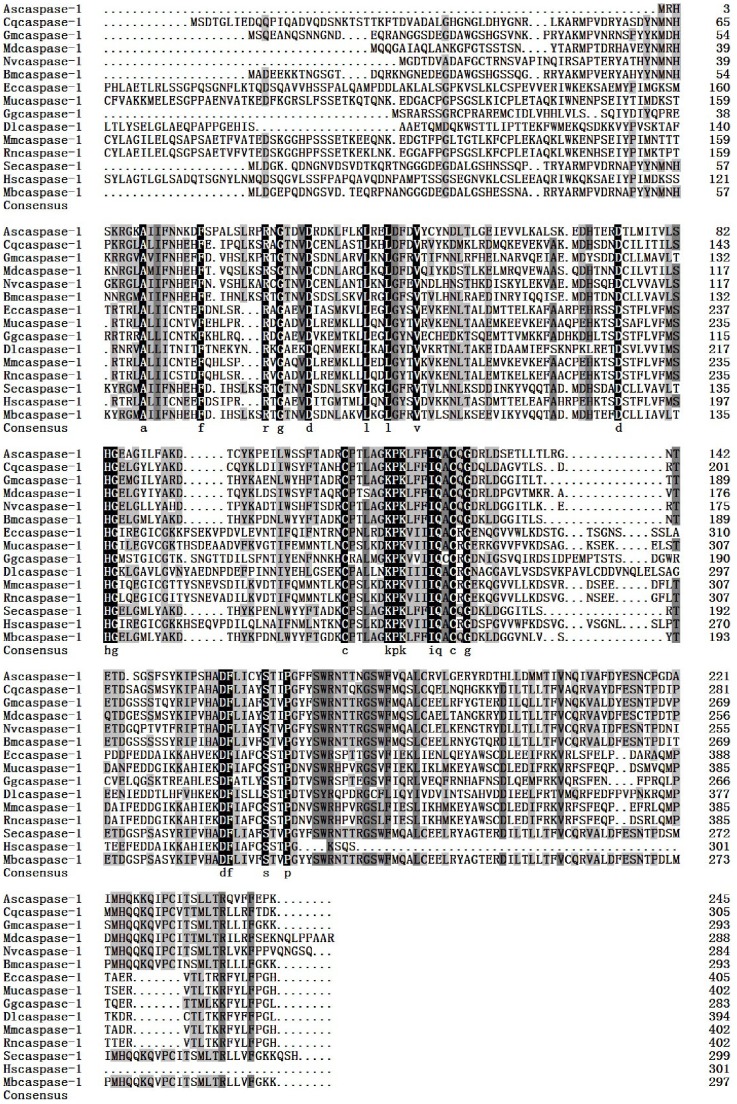
Multiple sequence alignment of the As-Caspase-1 protein. Sequence alignment of known *caspase-1* isoforms from 14 species. Black shading indicates identical amino-acid residues. Gray shading indicates less conserved residues. Pale gray shading indicates somewhat similar residues. The sequences and the accession numbers of caspase-1 are as follows: Ascaspase-1,*Artemia sinica,*AGB84766.1; Mdcaspase-1, *Musca domestica*, ACF71490.1; Cqcaspase-1, *Culex quinquefasciatus*, XP_001842236.1; Hmcaspase-1, *Heliconius melpomene*, ACU11588.1; Dpcaspase-1, *Danaus plexippus*, EHJ68333.1; Gmcaspase-1, *Galleria mellonella*, AEH76885.1; Slcaspase-1, *Spodoptera litura*, BAM62940.1; Bicaspase-1, *Bombus impatiens*, XP_003487262.1; Cgcaspase-1, *Crassostrea gigas*, AEB54801.1; Dlcaspase-1, *Dicentrarchus labrax*, ABB05054.1; Rncaspase-1, *Rattus norvegicus*, NP_036894.2; Ggcaspase-1, *Gallus gallus*, AAC69917.1; Sscaspase-1, *Sus scrofa*, NP_999327.1; Sacaspase-1, *Sparus aurata*, CAM32183.1;Hacaspase-1, *Helicoverpa armigera,* ABO93468.1; Cicaspase-1, *Ciona intestinalis*, XP_002129655.1; Bmcaspase-1, *Bombyx mori*, NP_001037050.1.

**Figure 6:**
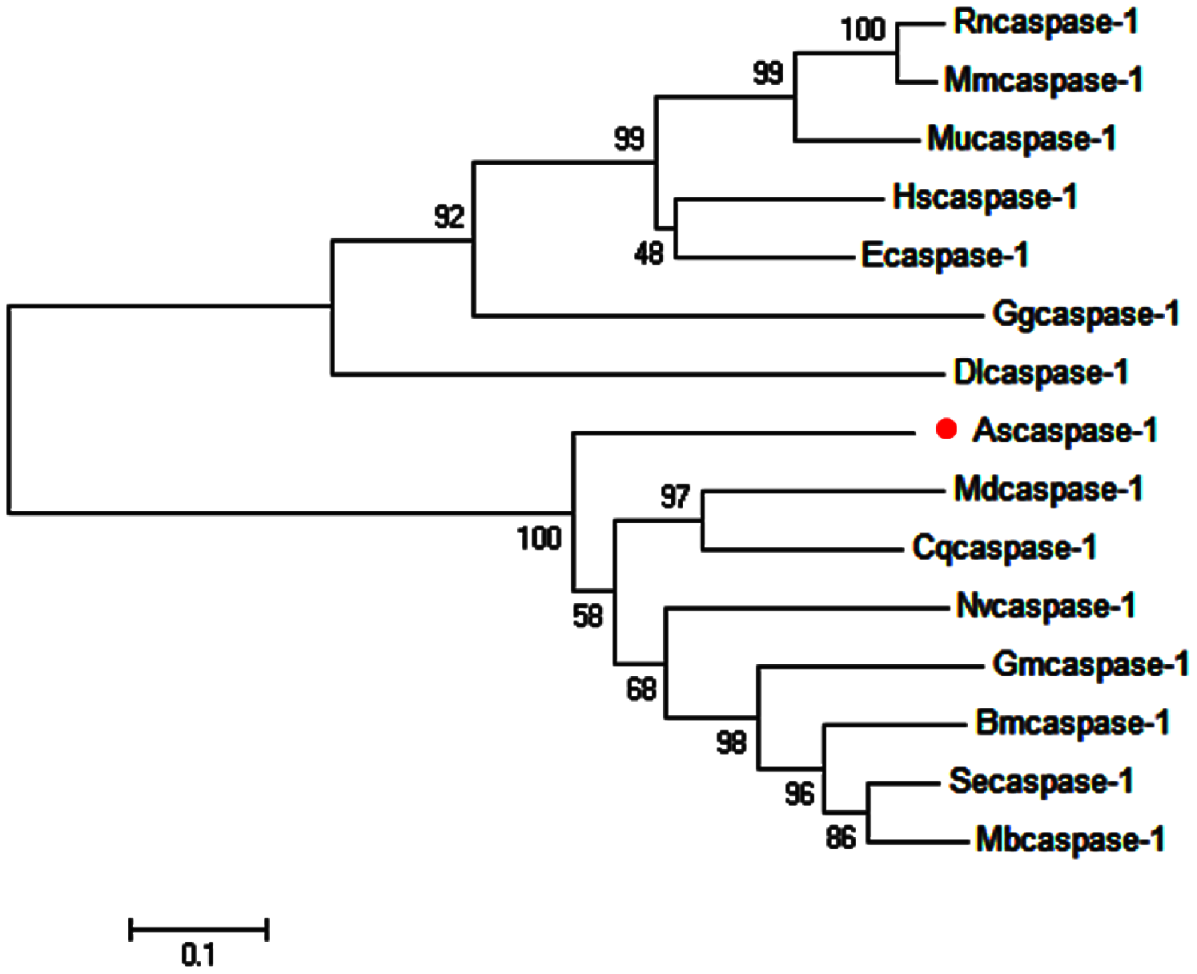
The neighbor-joining phylogenetic analysis of Caspase-1 protein. Phylogenetic tree of aligned amino acid sequences of *caspase-1* from *A. sinica* and 14 other species. A neighbor-joining phylogenetic tree was constructed using MEGA4.0, based on the sequences from *A. sinica* (this study) and 14 other species from GenBank. The sequences and their caspase-1 accession numbers are shown in Fig. 5. A red circle (•) indicates *As-caspase-1* from *A. sinica*.

The full length cDNA of the *A. sinica caspase-1* gene was in Fig. 4. SignalP3.0 analysis showed that *As*-Caspase-1 had no signal peptide. Secondary structure prediction demonstrated that the protein secondary structure comprised an Alpha helix (Hh; 23.47%), an Extended strand (Ee; 22.86%) and a Random coil (Cc; 53.47%). This protein has no transmembrane domain. Hydrophobicity analysis indicates that Caspase-1 is mostly hydrophilic. Caspase-1 has nine predicted serine phosphorylation sites, nine predicted threonine phosphorylation sites and two predicted tyrosine phosphorylation sites. Bioinformatic analysis suggests that Caspase-1 had the highest sequence homology (68.6% identity) with Caspase-1 from *Musca domestica* (Fig. 5). The Caspase-1 sequences of 15 species were selected to construct a phylogenetic tree (Fig. 6), which showed two main clusters: vertebrates and arthropods, with As-Caspase-1 clustered within the arthropods. The relationships displayed in the phylogenetic tree corresponded to their taxonomic classification.

**Figure 7:**
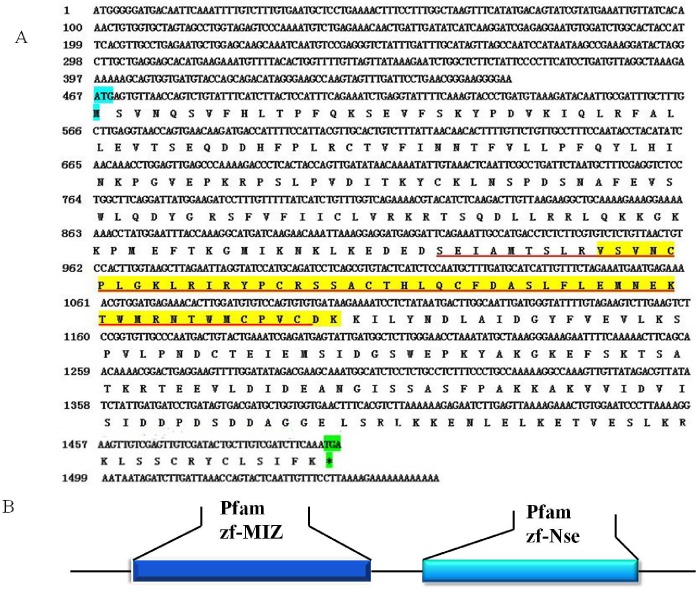
Nucleotide and deduced amino acid sequences of *As-sumo ligase* and putative protein domain. Sequence analysis of the cDNA and predicted peptide sequences of *As-sumo ligase*. The start codon is indicated in blue; the stop codon is indicated in green. The region defined by a straight red line shows the zf-MIZ domain. The zf-Nse domain is indicated in yellow. B. Result of domain analysis of putative As-sumo ligase protein. The mature putative protein includes a zf-MIZ domain and a zf-Nse.

**Figure 8:**
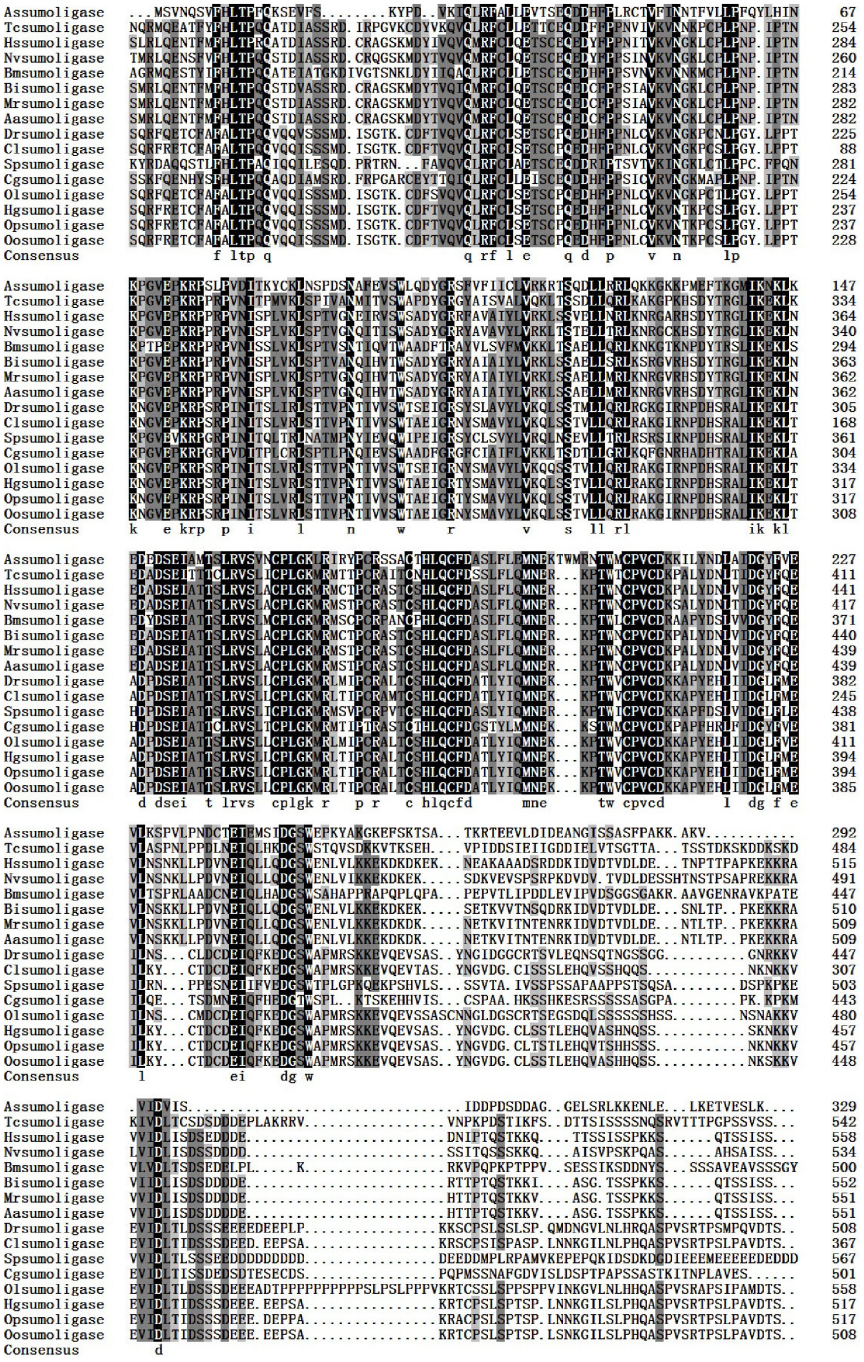
Multiple sequence alignment of the As-Sumo ligase protein. Sequence alignment of known sumo ligase isoforms from 15 species. Black shading indicates identical amino-acid residues. Gray shading indicates less conserved residues. Pale gray shading indicates somewhat similar residues. The sequences and the accession numbers of sumo ligase are as follows: Assumo ligase, *Artemia sinica,* AGO51518.1; Tcsumo ligase, *Tribolium castaneum, XP_974023.2*; Hssumo ligase, *Harpegnathos saltator*, EFN82639.1; Nvsumo ligase, *Nasonia vitripennis*, XP_003428228.1; Bmsumo ligase, *Bombyx mori*, XP_004922442.1; Bisumo ligase, *Bombus impatiens*, XP_003485893.1; Mrsumo ligase, *Megachile rotundata*, XP_003706512.1; Aasumo ligase, *Aedes aegypti*, XP_003706512.1; Drsumo ligase, *Danio rerio*, XP_692921.2; Clsumo ligase, *Columba livia*, EMC81308.1; Spsumo ligase, *Strongylocentrotus purpuratus*, XP_783836.3; Cgsumo ligase, *Crassostrea gigas*, EKC30788.1; Olsumo ligase, *Oryzias latipes*, XP_004067009.1; Hgsumo ligase, *Heterocephalus glaber*, XP_004855644.1; Opsumo ligase, *Ochotona princeps*, XP_004578008.1; Oosumo ligase, *Orcinus orca*, XP_004276281.1.

**Figure 9:**
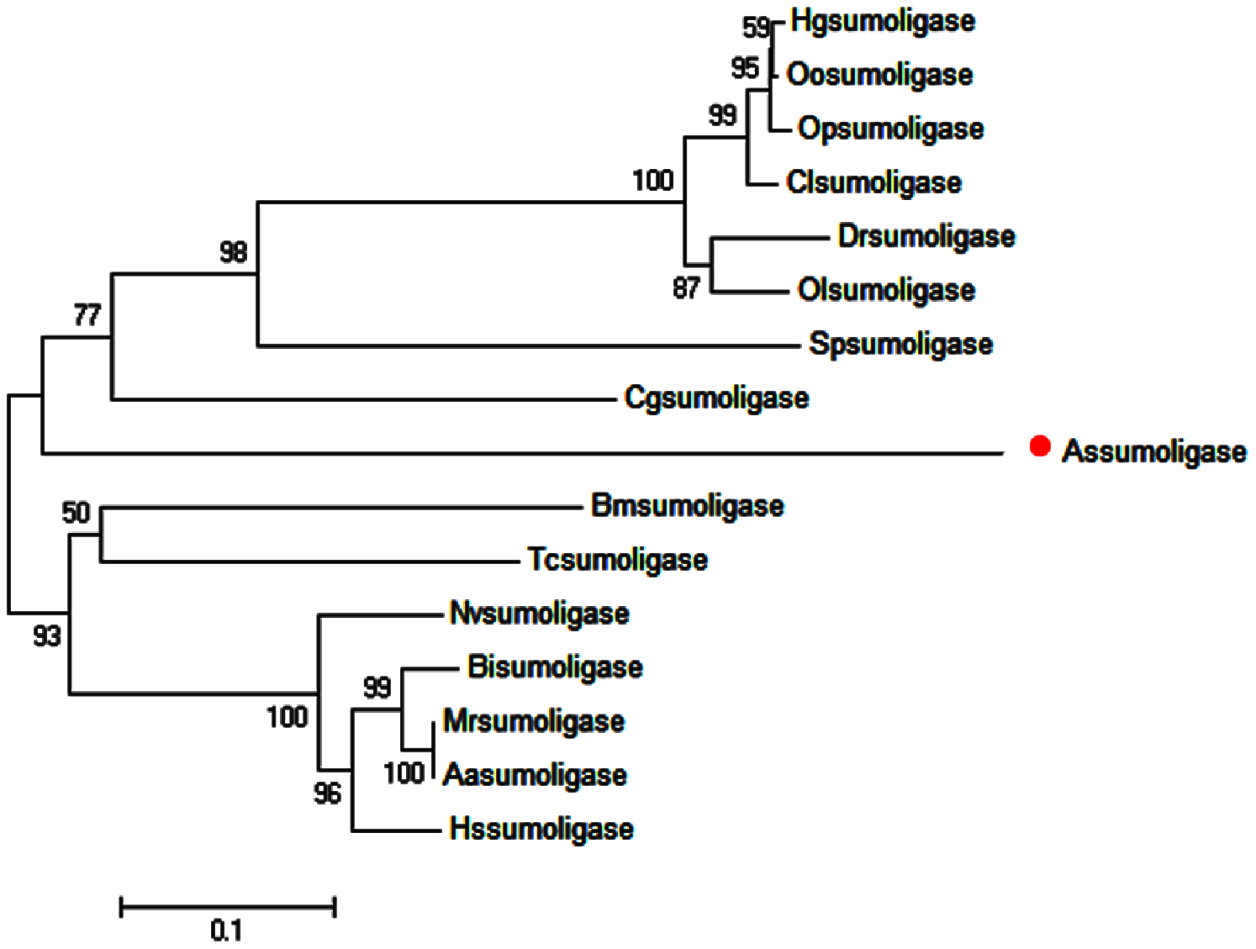
The neighbor-joining phylogenetic analysis of Sumo ligase protein. Neighbor-joining phylogenetic tree based on the amino acid sequences of *As-sumo ligase* and 15 other species from GenBank, using the sequence analysis tool MEGA 4.0. The sequences and their accession numbers are indicated in the legend of Fig. 8. A red dot indicates *As-sumo ligase* from *A. sinica*.

The full-length cDNA of the *A. sinica sumo ligase* gene was in Fig. 7. SignalP3.0 analysis showed that *As-*sumo ligase had no signal peptide. Secondary structure prediction demonstrated that the protein secondary structure included an Alpha helix (Hh; 26.24%), an Extended strand (Ee; 23.32%) and a Random coil (Cc; 50.44%). This protein has no transmembrane domain. Hydrophobicity analysis indicates that sumo ligase is slightly hydrophilic. sumo ligase was predicted to comprise fifteen serine phosphorylation sites, four threonine phosphorylation sites and three tyrosine phosphorylation sites. Bioinformatic analysis suggested that sumo ligase has the highest sequence homology (55% identity) with the sumo ligase from *Culex quinquefasciatus* (Fig. 8). The sumo ligase sequences of 15 species were selected to construct a phylogenetic tree (Bootstrapping = 1000), which showed three main clusters: vertebrates, arthropods and low-grade arthropods, with *As-*sumo ligase clustered within the arthropods. The relationships displayed in the phylogenetic tree corresponded to their taxonomic classifications (Fig. 9).

**Figure 10:**
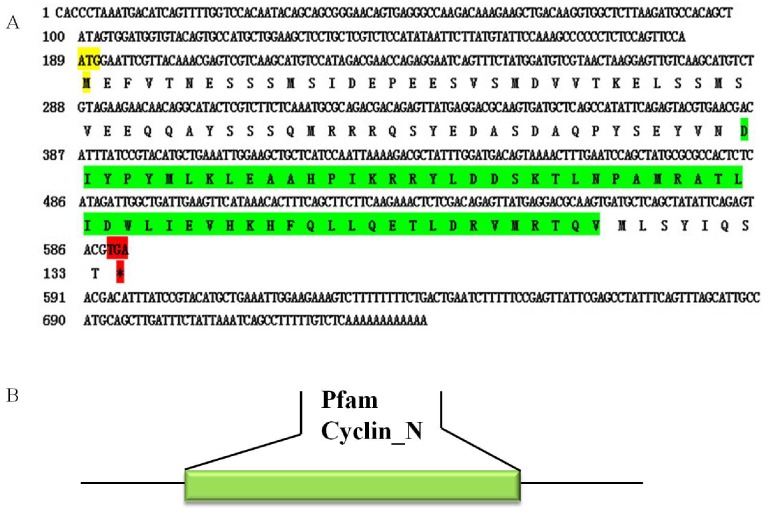
Nucleotide and deduced amino acid sequences of *As-cyclin B* and putative protein domain. Sequence analysis of the cDNA and predicted peptide sequences of *As-cyclin B*. The start codon is indicated in yellow; the stop codon is indicated in red; The region indicated in green shows the cyclin_N domain. B. Result of domain analysis of putative As-cyclin B protein. The mature putative protein includes a cyclin_N domain.

**Figure 11:**
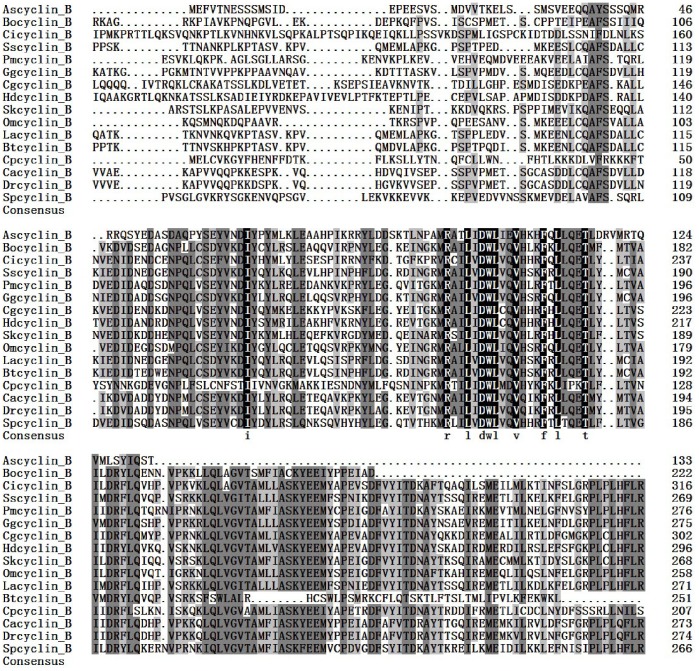
Multiple sequence alignment of the As-Cyclin B protein. Sequence alignment of known cyclin B isoforms from 15 species. Black shading indicates identical amino-acid residues. Gray shading indicates less conserved residues. Pale gray shading indicates somewhat similar residues The sequences and the accession numbers of cyclin B are as follows: Ascyclin B, *Artemia sinica,* KF149987.1; Bocyclin B, *Bombina orientalis*, ACJ12072.1; Cicyclin B, *Ciona intestinalis*, XP_002126215.2;Sscyclin B, *Sus scrofa*, NP_001107754.1; Pmcyclin B, *Penaeus monodon*, ACH72068.1; Ggcyclin B, *Gallus gallus*, NP_001004369.1; CgcyclinB, *Crassostrea gigas*, EKC39097.1; Hdcyclin B, *Haliotis diversicolor supertexta*, ADP06655.1; Skcyclin B, *Saccoglossus kowalevskii*, NP_001158480.1; Omcyclin B, *Oncorhynchus mykiss*, NP_001118131.1; Lacyclin B, *Loxodonta Africana*, XP_003418445.1; Btcyclin B, *Bos Taurus*, AAX46547.1; Cpcyclin B, *Cryptomonas paramecium*, XM_003239530.1; Cacyclin B, *Carassius auratus*, EU333815.1; Drcyclin B, *Danio rerio*, AF268043.1; Spcyclin B, *Scylla paramamosain*, FJ595022.1.

**Figure 12:**
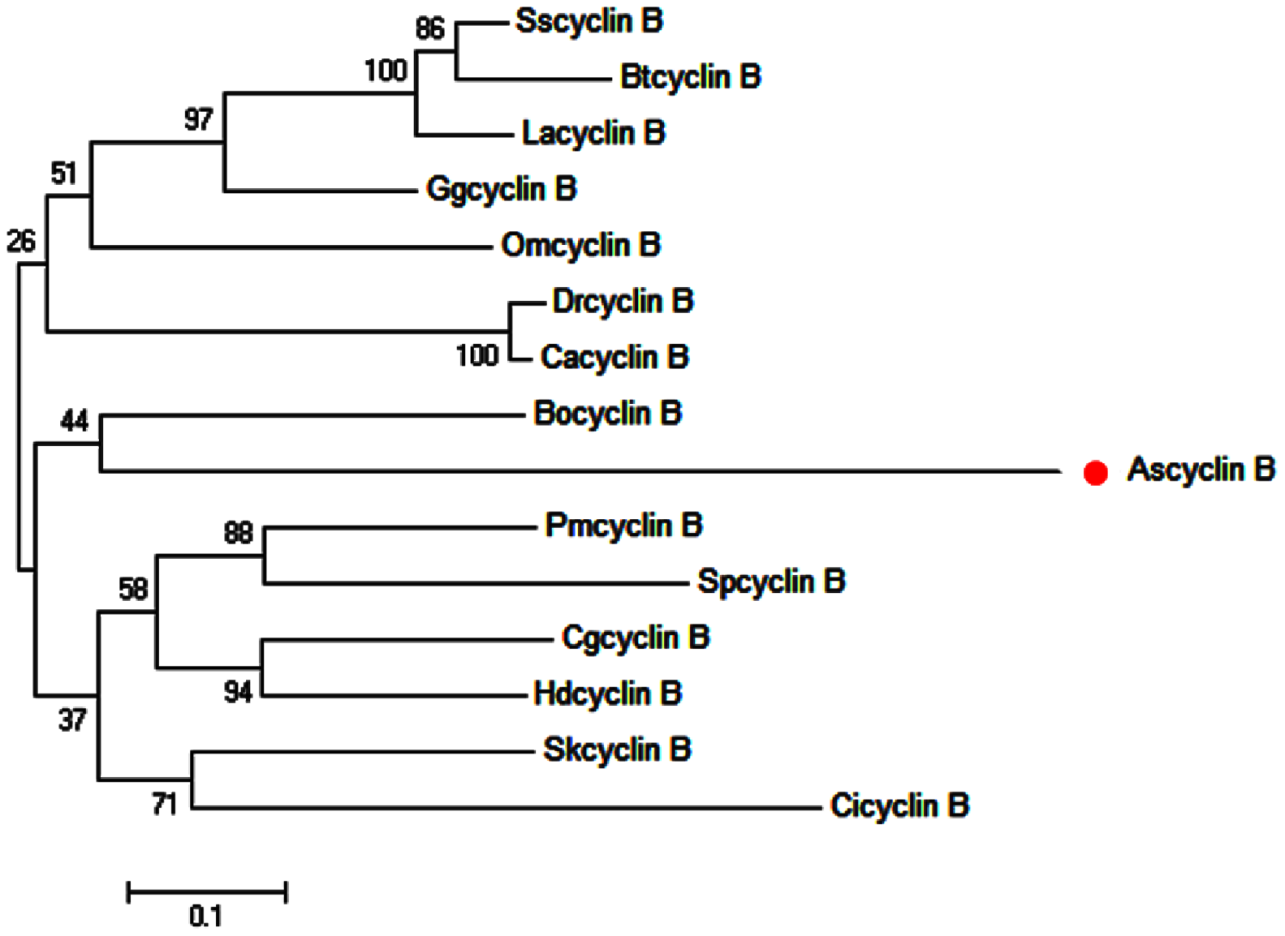
The neighbor-joining phylogenetic analysis of Cyclin B protein. Phylogenetic tree of aligned amino-acid sequences of cyclin B from *Artemia sinica* and 14 other species. A neighbor-joining phylogenetic tree was constructed using cyclin B sequences from *A. sinica* and 15 other sequences in GenBank, using the sequence analysis tool MEGA 4.1. The sequences and their accession numbers are indicated in the legend of Fig. 11. Circle (•) indicates *cyclin B* from *A. sinica*.

The full length cDNA of *A. sinica cyclinB* gene was in Fig. 10. SignalP3.0 analysis showed that *As-*Cyclin B had no signal peptide. Secondary structure prediction demonstrated that the protein secondary structure included an Alpha helix (Hh; 55.64%), an Extended strand (Ee; 3.76%) and a Random coil (Cc; 40.6%). This protein had no transmembrane domain. Hydrophobicity analysis indicated that Cyclin B is mostly hydrophilic. Cyclin B was predicted to have sixteen serine phosphorylation sites, eleven threonine phosphorylation sites and a tyrosine phosphorylation site. Bioinformatics analysis suggested that Cyclin B had the highest sequence homology (76% identity) with Cyclin B from *Bombina orientalis* (Fig. 11). The Cyclin B sequences of 16 species were selected to construct a NJ phylogenetic tree (Bootstrapping = 1000) (Fig. 12), which showed three main clusters: vertebrates, arthropods and nematodes, with *As-*Cyclin B clustered within the arthropods. The relationships displayed in the phylogenetic tree corresponded to their taxonomic classifications.

### Expression Pattern of *As-sumo-1, As-caspase, As-sumo Ligase* and *As-cyclin B*

**Figure 13:**
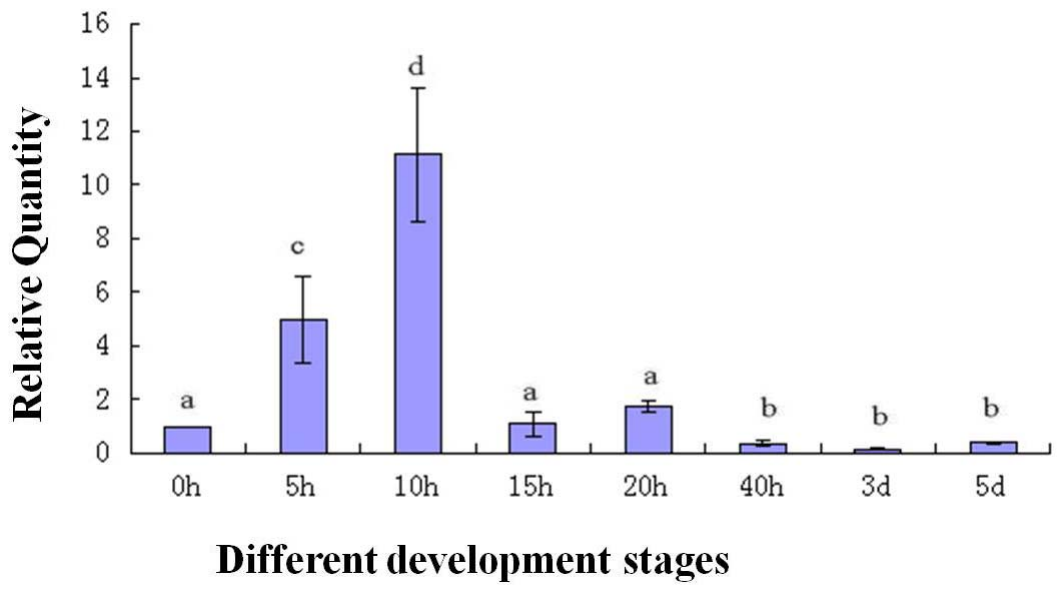
Real-time quantitative PCR analysis of *As-sumo-1* expression during different stages of *Artemia sinica* development. The expression of *sumo-1* was measured at various time points during development. The x-axis indicates the developmental stage (0 h–5 d); the y-axis indicates the expression level relative to expression at 0 h. Data are the means ± SD of triplicate experiments. Significant differences between developmental stages (*P*<0.05) were analyzed by one-way analysis of variance (ANOVA) and are indicated with letters (a, b, c and d).

**Figure 14:**
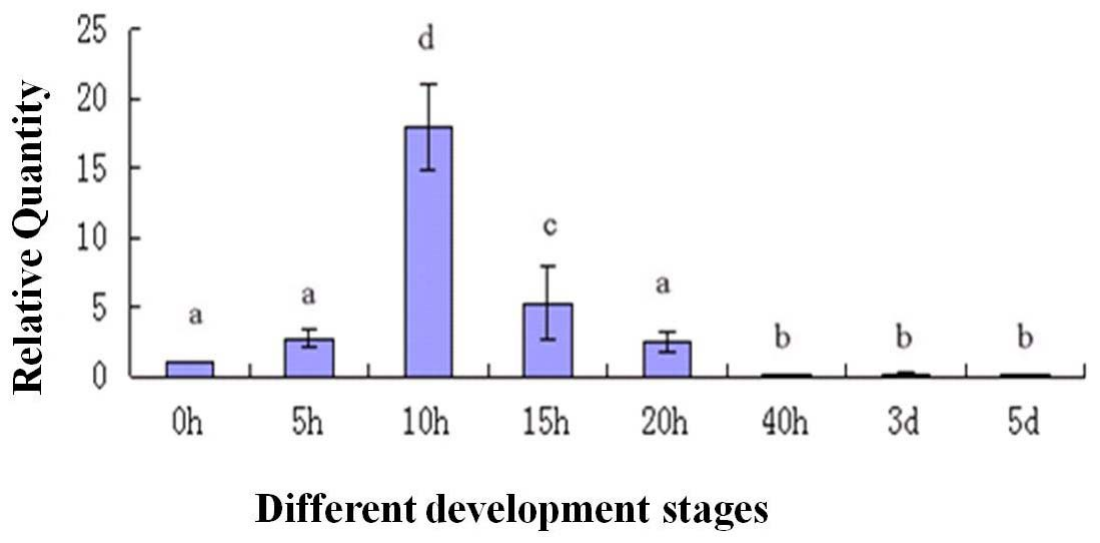
Real-time quantitative PCR analysis of *As-caspase-1* expression during different stages of *Artemia sinica* development. The expression of *As-caspase-1* was measured at various time points during development. The x-axis indicates the developmental stage (0 h–5 d); the y-axis indicates the expression level relative to the expression level at 0 h. Data are the means ± SD of triplicate experiments. Significant differences between developmental stages (*P*<0.05) were analyzed by one-way analysis of variance (ANOVA) and are indicated with letters (a, b, c and d).

**Figure 15:**
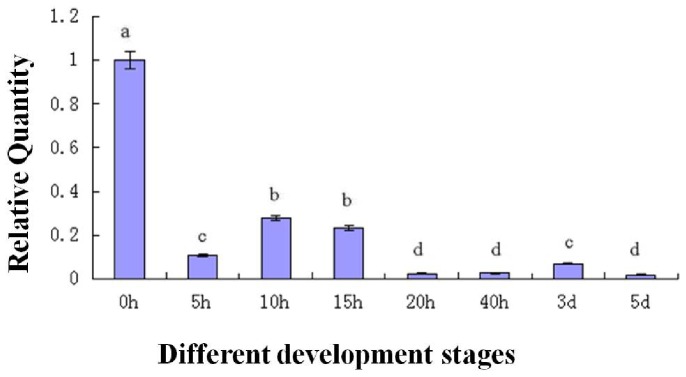
Real-time quantitative PCR analysis of *As-sumo ligase* expression during different stages of *Artemia sinica* development. The expression of *As-sumo ligase* was measured at various time points during development. The x-axis indicates the developmental stage (0 h–5 d); the y-axis indicates the expression level relative to the expression level at 0 h. Data are the means ± SD of triplicate experiments. Significant differences between developmental stages (*P*<0.05) were analyzed by one-way analysis of variance (ANOVA) and are indicated with letters (a, b, c and d).

**Figure 16:**
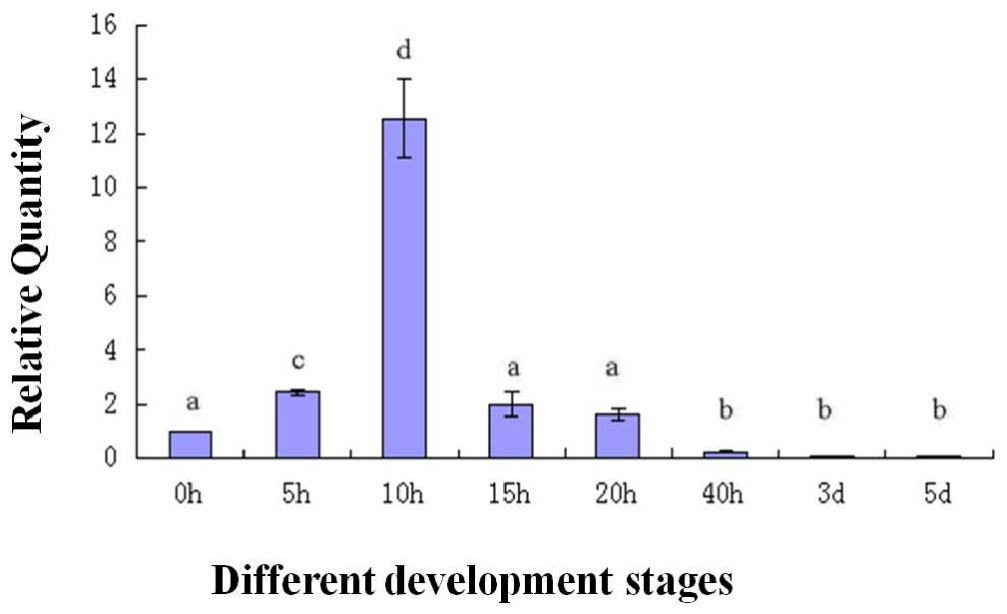
Real-time quantitative PCR analysis of *As-cyclin B* expression during different stages of *Artemia sinica* development. The expression of *As-cyclin B* was measured at various time points during development. The x-axis indicates the developmental stage (0 h–5 d); the y-axis indicates the expression level relative to the expression level at 0 h. Data are the means ± SD of triplicate experiments. Significant differences between developmental stages (*P*<0.05) were analyzed by one-way analysis of variance (ANOVA) and are indicated with letters (a, b, c and d).

To determine the expression of the *As-sumo-1* transcript during embryonic development in *A. sinica*, real-time PCR analysis was performed (Fig. 13). The relative expression level of *As-sumo-1* transcript increase from 0 h to 15 h, followed by a decreased at 20 h until 5 d. A similar expression pattern was observed for *As-caspase-1* (Fig. 14), *sumo ligase* (Fig. 15) and *cyclinB* (Fig. 16). The trend of *caspase-1* and *cyclin B* expression were similar to *sumo-1*, but the expression of *sumo ligase* at 0 h was higher than the others.

### Prokaryotic Expression of *As*-SUMO-1 Protein and Production of a SUMO-1 Antibody

**Figure 17:**
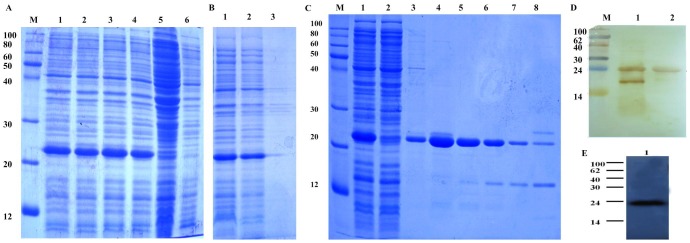
The results of prokaryotic expression of *As-*SUMO-1 like protein. (A) Expression of *Artemia sinica As-*SUMO-1 recombinant protein. M: protein markers from 12–100 kDa. Lanes 1–4 show the expression of *As*-SUMO-1 recombinant protein from four induction treatments (1 mM IPTG at 37°C, 1 mM IPTG at 30°C, 0.25 mM IPTG at 37°C, and 0.25 mM IPTG at 30°C, respectively). The arrow shows the position of the expressed recombinant protein. Lane 5: total proteins from non-induced cells. Lane 6: total proteins from induced cells harboring pET-30a (control). (B) Detection of soluble *Artemia sinica As*-SUMO-1 recombinant protein. Lane 1: total proteins from induced cells harboring pET-30a-sumo-1. Lane 2: soluble fraction of the lysate from induced cells harboring pET-30a-sumo-1. Lane 3: insoluble fraction of the lysate from induced cells harboring pET-30a-sumo-1. (C) Purification of recombinant *Artemia sinica As*-SUMO-1 protein. M: protein markers from 12–100 kDa. Lane 1: total proteins extracted from induced cells harboring pET-30a-sumo-1. Lane 2: flow-through eluate of total proteins. lanes 3–8: column elution with elutant containing 20 mM, 40 mM, 60 mM, 80 mM,100 mM and 300 mM imidazole, respectively. (D) Detection of the His-tag in the purified protein. M: protein markers from 14–100 kDa. Lane 1: total proteins from induced cells harboring pET-30a-SUMO-1. Lane 2: purified recombinant pET-30a-SUMO-1 protein. (E) Western blot showing specific binding of the antibody to the purified protein.

*As*-SUMO-1 protein was prokaryotically expressed, purified and its molecular weight determined as 18 kDa. SDS–PAGE analysis revealed that the recombinant protein was expressed under all four induction conditions (Fig. 17A). The fourth treatment (0.25 mM IPTG at 30°C) was chosen for further research. SDS–PAGE analysis showed that the recombinant protein was present in the soluble fraction isolated from *E. coli* BL21 (Fig. 17B). A relatively pure protein was obtained after purification and dialysis (Fig. 17C). The product was checked by western blotting with a mouse antibody directed against the His-tag as the primary antibody (Fig. 17D). The molecular mass of the recombinant protein was larger than 18 kDa, which could be explained by the basic amino acids of the His-tag causing the protein to migrate more slowly on SDS–PAGE. After adding adjuvants to the purified protein and immunizing rabbits, we acquired a polyclonal antibody of rabbit with a valence of 1∶500000 as determined by an enzyme linked immunosorbent assay (ELISA). Western blotting using the prepared rabbit antibody showed that the antibody could specifically bind to the purified protein (Fig. 17E).

### Expression of *As-*SUMO-1, *As*- Caspase-1, *As*-Mdm2, *As*-p53, *As*-Cyclin E and *As*-Cyclin B Protein at Different Developmental Stages

**Figure 18:**
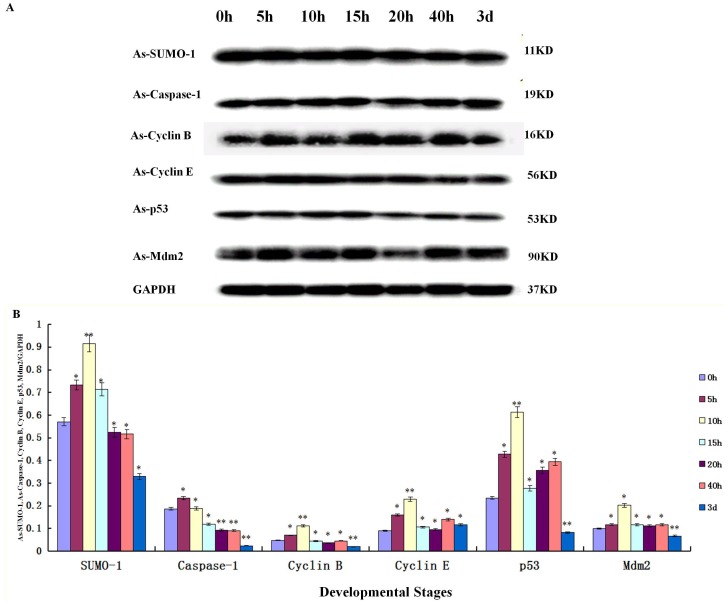
Western blot analysis of *As*-SUMO-1, *As*-Caspase-1, *As*-Mdm2, *As*-p53, *As*-Cyclin E, *As*-Cyclin B. (A) Western blot showing the expression of *As*-SUMO-1, *As*-Caspase-1, *As*-Mdm2, *As*-p53, *As*-Cyclin E, *As*-Cyclin B protein at different developmental stages in *A. sinica*. The band intensities for these proteins were normalized against the GAPDH protein. (B) Values are expressed as arbitrary units of relative value. The expression of these proteins at 0 h was used as the reference, and asterisks indicate statistically significant differences.

The expression level of *As*-SUMO-1 at different developmental stages in *A. sinica* revealed an upward trend during early development from 0 h to 10 h, and a gradual downward trend after breaking the shell (Fig. 18). The expression trend of Caspase-1, Cyclin B, Cyclin E, p53 and Mdm2 were similar to SUMO-1.

### Localization of *As*-SUMO-1 Expression at Different Developmental Stages

**Figure 19:**
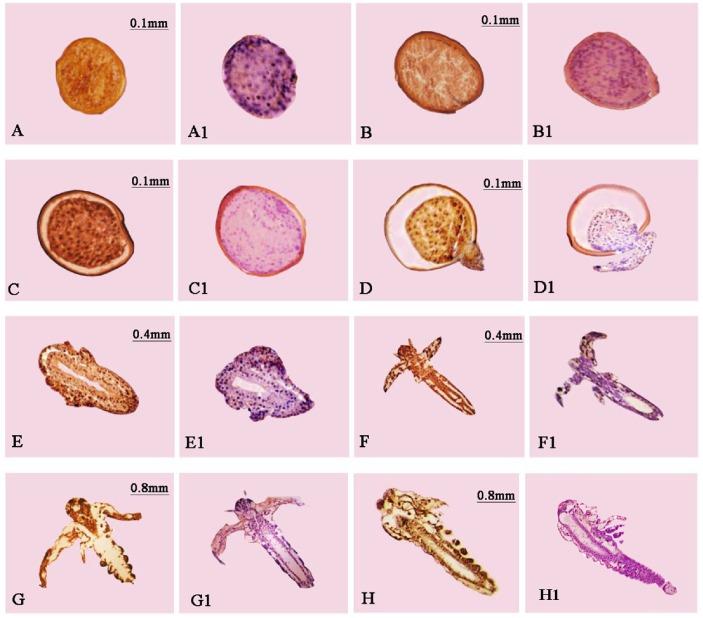
Immunohistochemical analysis of the expression of *As*-SUMO-1 at different developmental stages in *Artemia sinica*. A–H represent experimental groups and A 1-H 1 represent the control groups. (A) 0 h, gastrula stage of *Artemia* cysts; (B, C, and D) 5 h, 10 h, 15 h, embryonic stage; (E and F) 20 h and 40 h, nauplius stage; (G) 3 d, metanauplius larval stage; (H) 5 d, pseudoadult stage.

Immunohistochemistry showed that at the 0 h embryo stage, *As*-SUMO-1 was expressed throughout the cyst of *A. sinica* (Fig. 19A), and this situation continued until the 10 h embryo stage (Fig. 19B, C). At 15 h, the cyst shells were cracked, exposing the nauplius’s head with the tail remaining inside, which is usually referred to as the umbrella stage (Fig. 19D), at this stage, the *As*-SUMO-1 was expressed from the head to the tail. At the 20 h stage, expression of *As*-SUMO-1 was detected throughout the whole body (Fig. 19E). The cephalothorax and abdomen began to differentiate and appendages appeared at 40 h (Fig. 19F); *As*-SUMO-1 was detected in these regions. At 3 d and 5 d, *As*-SUMO-1 appeared in the regions of the enteron and external appendages of the polypide (Fig. 19G, H). The negative control (no incubation with the anti-*As*-SUMO-1 antibody) showed no positive signals.

### siRNA Assay

**Figure 20:**
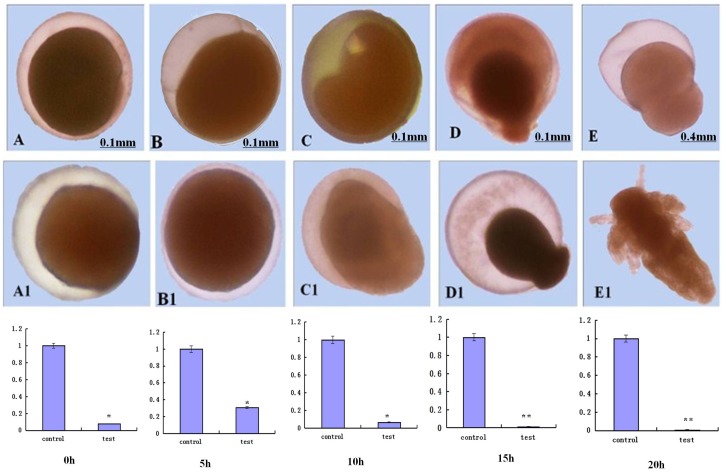
The relative level of *sumo-1* mRNA expression in larvae soaked with dsRNAs for different times. *sumo-1*-RNAi depleted expression of *As-sumo-1* at different developmental stages from 0 h to 20 h in *Artemia sinica*. A–E represent experimental groups treated with *sumo-1*-RNAi and A1-E1 represent the control groups.

In the *As-sumo-1* siRNA knockdown experiments, deformations appeared at the nauplius stage (15 h–20 h). The experimental individuals grew and moved more slowly than the controls. The rate of death and of deformation both increased. The mortality of experimental group was 31.63%, and the mortality of control group was 31.08%. The difference between the two groups were non-significant. If experimental group continue to develop, the deformity individuals all continue to survive. To determine the amount of *As-sumo-1* transcription during embryonic development of *A. sinica* after siRNA knockdown, real-time PCR analysis was performed. At 0 h, 5 h, 10 h, 15 h and 20 h, the expression of *As-sumo-1* mRNA was lower in all experimental individuals compared with the control group (Fig. 20).

## Discussion

In this study, we isolated the full-length cDNA sequences of *sumo-1*, *caspase-1*, *sumo ligase*, *cyclin B* in *A. sinica* for the first time. *As-sumo-1* gene encodes a putative protein of 92 amino acid with a highly conserved UBQ domain. *As-sumo-1* lacks a signal peptide and transmembrane regions and was expressed was primarily in the cytoplasm, mitochondria and nucleus, indicating that SUMO-1 is conjugated to numerous intracellular targets and serves to modulate protein interaction, localization, activity or stability, including nucleocytoplasmic transport [17]. PIAS (Protein inhibitor of activated STATs) proteins, serving as E3-like ligases, stimulate the attachment of SUMO to target proteins. SUMO modification, like Ubiquitin-mediated proteolysis, is involved in the regulated turnover of proteins required for controlling cell cycle progression.

SUMO-1 has a highly conserved ψ KXE/D motif, KXD/E, although recent studies have also indicated roles for modifications at noncanonical lysine residues [18] [19] [20], the C-terminal double Gly-Gly residues for conjugation and a conserved ubiquitin domain in all species [21]. The phylogenetic tree revealed that UBQ-containing proteins are highly conserved in *A. sinica* and other species, especially closely-related invertebrates, such as *Artemia franciscana*, *Penaeus monodon* and *Apis mellifera*. The sequence alignment showed a high similarity between *A. sinica* and arthropods. *As-sumo ligase*, a kind of E3 ligase, has a MIZ/SP–RING zinc finger domain. This domain has SUMO ligase activity and is involved in DNA replication and repair, chromosome organization, mitosis, and transcriptional regulation [22] [23]. *As-sumo ligase* lacks a signal peptide and transmembrane regions, and its expression was primarily confined to the nucleus and cytoplasm. *As-caspase*-1 lacks a signal peptide and transmembrane regions, and its expression was confined to the cytoplasm, mitochondria and nucleus. *As-cyclin B*, like other cyclins, is rich in alpha helices (55.64%), especially in the N-terminal domain. This protein has no transmembrane domain, is mostly hydrophilic and has no signal peptide.

Using real-time PCR, we determined that *As-sumo-1* is highly expressed in *A. sinica* from 0 h to 10 h. As the diapause embryo becomes activated, *As-sumo-1* expression gradually increased, which suggested that SUMO-1 is associated with the cell cycle [24].

As the embryos left the stable embryonic environment and come into contact with highly saline water, the expression level of *sumo-1* remained a basal level. From 0 h to 15 h, the embryos progress from cyst to nauplius, and the embryonic cells may experience cell division and synthesize proteins that are necessary for embryonic activities. During this phase expression of *sumo-1* increased. During development, which is accompanied by cell differentiation, the expression of *As-sumo-1* gradually decreased. During post-embryonic development (from 3 d to 5 d), *As-sumo-1* expression decreased significantly, as organ growth is nearly complete. During these stages of development, body cell apoptosis occurs, accompanied by downregulation of *As-sumo-1* expression. Therefore, *As-sumo-1* expression may be maintained at a low level in adults. The pattern of expression of *sumo ligase* was similar. Conjugation of SUMO to its substrates occurs through an enzymatic cascade composed of an E1 activating enzyme (SAE1/UBA2) [25], an E2 conjugating enzyme (UBC9) and E3 protein ligases, which results in the formation of an isopeptide bond between the C-terminus of SUMO and the ε-amino group of a substrate lysine residue [26]. E1, E2 and E 3 are the activating, conjugating and SUMO ligase enzymes of the conjugation pathway, respectively. Thus at 0 h, the expression of E3 *sumo ligase* was high.

Members of the Caspase family play a central and evolutionary role in apoptosis, which removes the unwanted, damaged and dangerous cells during development to maintain homeostasis. Caspase-1 (interleukin-1 b converting enzyme), which functions in the production of proinflammatory cytokines and in apoptosis [28] [29] [30], is a transcriptional target of p53 [27]. Caspase-1 knockout mice are developmentally normal, but are defective in the production of mature cytokines interleukin-1b and interleukin-18. These mice are resistant to septic shock and show a partial defect in apoptosis [31]. Caspase-1 is initially expressed as an inactive precursor. Caspases play important roles in apoptosis signaling and effector mechanisms [32]. The rate of cell division of *A. sinica* from 0 h to 10 h went up, the worm were in organ differentiation, along with spontaneous apoptosis process. From 15 h to 5 d, the cells of the polypide are dividing, and the expression of *As*-*caspase-1* was downregulated. In the current study, the pattern of *As-caspase-1* expression was similar to that of *As-sumo-1*.

The rapid cycling from interphase to mitosis in early embryos follows the activity of the highly conserved maturation-promoting factor. Maturation-promoting factor consists of a regulatory subunit, cyclin B, and a catalytic subunit, cdk1. Activation of cdk1 is dependent on it binding to its regulatory subunit, cyclin B, and subsequent phosphorylation events. Levels of cyclin B begin to rise during G2 [31] [32]. In the current study, the pattern of *As-cyclin B* expression was similar to that of *As-sumo-1*.

Previous expression localization studies showed that SUMO-1 has a wide range of expression in crab [33]. Our study of the spatial expression of *As*-SUMO-1 at eight developmental stages in *A. sinica* showed that the distribution of *As*-SUMO-1 was extensive. It was present in almost all body parts and during every developmental stage. Sumoylation plays important roles in essential cellular processes, including the cell cycle; thus, *As*-SUMO-1 activity of cells occurred throughout the lifecycle of *Artemia sinica*. Thus, it is unsurprising that SUMO-1 should be widely distributed; however, *As*-SUMO-1 was expressed at high levels at 0 h, 5 h and 10 h in the body of *A. sinica*. The cell cycle plays a central role in controlling the rate of cell division [34]. Cell division was very active from 0 h to 10 h, regulating processes during meiosis and mitosis [35], which suggests that sumoylation is highly active during mitosis and meiosis.

Much evidence has indicated that SUMO is essential for many important cellular events, including DNA replication and repair, kinetochore-tubulin attachment, genome integrity maintenance [24]. SUMO is synthesized as a larger precursor that must be processed to reveal the C-terminal glycine residue that is linked to lysine side chains in target proteins. The expression level of *As*-SUMO-1 at different development stages in *A. sinica* showed an upward trend during early development, reaching its highest level at 10 h, before showing a gradual downward trend from 15 h to 3 d. The expression of Caspase-1, Cyclin B, p53, Cyclin E, Mdm2 all showed similar expression patterns. Embryos enter the diapause stage because of a lack of nutrients in the environment or some other adverse conditions. The 0 h cysts were incubated in seawater at a suitable temperature and light to break diapause; during this period the expression of *As*-SUMO-1 was low and only increased when the cysts resumed development in a favorable environment. During this period from 0 h to 10 h, the rate of mitosis and SUMO-mediated degradation increased. SUMO-1, Cyclin E and Cyclin B are cell cycle-related proteins. However, p53, Caspase-1 and Mdm2 are apoptosis-related proteins. From 0 h to 10 h, there was considerable cellular activity in the cysts, including cell division and apoptosis. Along with cell division, there must be apoptosis. These processes are necessary for maintaining the essential reserve; accordingly, the expressions of SUMO-1, Cyclin E, Cylin B, p53 and Mdm2 showed an upward trend. p53 mainly acts as an inducible and sequence-specific transcription factor on genes whose products either inhibit cell-cycle progression or induce apoptosis [36]. To avoid unwanted cell death or cell cycle arrest, the activities of p53 have to be regulated in unstressed cells or cells that have completed DNA repair. Mdm2, a RING-type ligase, is itself a target gene of p53 and its levels are therefore particularly elevated during the late stages of the DNA damage response. Thus, Mdm2-mediated polyubiquitination and nuclear degradation may be important to limit p53 activity once DNA repair has been completed. In the body of *A. sinica* there should be few damaged cells; therefore, the expressions of p53 and Mdm2 were at a basal level. From 0 h to 5 h, the expression of p53 increased as did that of Mdm2. From 15 h to 3 d, the expression of p53 and Mdm2 increased as the larvae adapted to the external environmental conditions. At 3 d, the larvae had completely adapted to the environment, and the expression of p53 and Mdm2 decreased.

Cyclins are synthesized and accumulated during each cell cycle and then destroyed at the end of each round of mitosis, immediately preceding the metaphase-anaphase transition [37]. Cyclin E, a regulatory subunit of cyclin-dependent kinase 2 (Cdk2), is an important regulator of entry into S phase in the mammalian cell cycle. In normal dividing cells, Cyclin E accumulates at the G1/S-phase boundary and is degraded as cells progress through S phase [38], [39]. Appropriate regulation of cyclin E is critical for a number of cellular processes [40]–[43]. Cyclin B is a potent inducer of meiosis I. Cyclin B has all the properties required of an M phase inducer for meiosis I [36]. At 0 h to 10 h, the cells in the cysts were largely progressing through cell division and were in G1, S, G2, or M phase. Consequently, the expressions of Cyclin B and Cyclin E increased. From 15 h to 3 d,the cells began to differentiate during the development of larvae; therefore, the expression of Cyclin B and Cyclin E showed a downward trend, as did *As-*SUMO-1.

The protein expression of Caspase-1 mirrored its gene expression from 0 h to 10 h. The gene expression of *As-caspase-1* was highest at 10h, whereas the protein expression was highest at 5h. Caspases play important roles in apoptosis signaling and effector mechanisms [32]. From 0 h to 10 h, the cysts of *A. sinica* may need to synthesize certain proteins to prepare to regulate apoptosis, hence the highest expression of Caspase-1 at 5h While the larvae grew, the expression of Caspase-1 was downregulated, indicating lower levels of apoptosis in the larvae.

siRNA represents the process of double-stranded(ds) RNA-dependent, post-transcriptional gene silencing [44]. RNA interference reduces transcription, thereby reducing the levels of the target protein, which may have phenotypic effects. In our *sumo-1* knockdown experiments, increased mortality and malformation were observed in larvae expressing the siRNA targeting SUMO-1. Surviving individuals not only moved slower than the wild-type, but also developed slower than the wild-type. Most of the polypides with *sumo-1* knockdown died, indicating that SUMO-1 is an essential protein for the viability of *A. sinica.*

### Concluding Remarks

Our study indicated that SUMO-1 plays an important role in the cell cycle regulation and modification, along with Cyclin B, Cyclin E. Caspase-1, p53, Mdm2 play roles in cell apoptosis. SUMO also functions in transcriptional repression and differentiation. The balance of cell proliferation and apoptosis is vital to an organism’s development and function. During the development of *A. sinica*, cell cycle regulation and cell apoptosis act constantly. In this paper, we determined that SUMO-1 has an important role in linking cell cycle-related proteins and cell apoptosis-related proteins. From 0 h to 15 h, *A. sinica* cells undergo many activities, including cell cycle and cell apoptosis. The transcription levels of *caspase-1*, *cyclin B*, *sumo ligase* in *A. sinica* were similar to *As-sumo-1*. The protein levels of Caspase-1, Cyclin E, Cyclin B, p53 and Mdm2 were also similar to that of SUMO-1. *As*-SUMO-1 showed no tissue specificity during development. RNAi of *sumo-1* indicated that *sumo-1* is indispensible for growth and development of *A. sinica*. In conclusion, *As-*SUMO-1 is strictly required for the processes of diapause embryo resumption and early embryo development in *A. sinica*.
